# DNA Sensors’ Signaling in NK Cells During HHV-6A, HHV-6B and HHV-7 Infection

**DOI:** 10.3389/fmicb.2020.00226

**Published:** 2020-02-19

**Authors:** Daria Bortolotti, Valentina Gentili, Elisabetta Caselli, Mariangela Sicolo, Irene Soffritti, Maria D’Accolti, Isabel Barao, Antonella Rotola, Dario Di Luca, Roberta Rizzo

**Affiliations:** ^1^Department of Chemical and Pharmaceutical Sciences, University of Ferrara, Ferrara, Italy; ^2^Department of Medical Sciences, Section of Microbiology, University of Ferrara, Ferrara, Italy; ^3^School of Medicine, University of Nevada, Reno, Reno, NV, United States

**Keywords:** DNA sensors, human herpesvirus, natural killer cells, HHV-6A, HHV-6B, HHV-7

## Abstract

**Objectives:**

The host DNA sensor proteins TLR9, STING, IFI16 are central signaling molecules that control the innate immune response to cytosolic nucleic acids. Here we propose to investigate how Natural killer (NK) cell infection by human herpesvirus (HHV)-6A, HHV-6B or HHV-7 is able to modify DNA sensor signaling in NK cells.

**Methods:**

We infected the NK92 cell line and primary NK cells with cell-free inocula of HHV-6A, HHV-6B or HHV-7 and evaluated TLR9, STING, and IFI16 pathway expression by Real-Time PCR, Western Blot, immunofluorescence and flow cytometry for 1, 2, 3, and 6 days post-infection. We evaluated NK cell cytokine-producing by Real-Time PCR and enzyme immunosorbent assay.

**Results:**

NK92 and primary NK cells were promptly infected by three viruses, as demonstrated by virus presence (DNA) and transcription (RNA) analysis. Our data show STING/STAT6 up-modulation in HHV-6A infected NK cells. NK cells infected with HHV-6B and HHV-7 up-regulated CCL3, IFN-alpha, TNF-alpha, IL-8 and IFN-gamma and slightly induced IL-4, and CCL4. HHV-6A infected NK cells up-regulated IL-4 and IL-13 and slightly induced IL-10, TNF-alpha, IFN-alpha, and IFN-gamma.

**Conclusion:**

For the first time, we demonstrate that HHV-6A, HHV-6B, and HHV-7 infections have a differential impact on intracellular DNA sensors. HHV-6B and HHV-7 mainly lead to the active control of *in vivo* viral spreading by pro-inflammatory cytokine secretion via TLR9. HHV-6A infected NK cells conversely induced STING/STAT6 pathway, as a mechanism of anti-viral activation, but they were characterized by a Th2 type response and a non-cytotoxic profile, suggesting a potential novel mechanism of HHV-6A-mediated immunosuppression.

## Introduction

Three herpesviruses gaining medical interest are the human herpesvirus-6 (HHV-6) A and B and human herpesvirus-7 (HHV-7). They are members of the *Herpevirales* order, *Herpesviridae* family, *Betaherpesvirinae* subfamily, and *Roseolavirus* genus.

HHV-6, as HHV-6A and HHV-6B are commonly called when they are not separated into two species, has a wide cell tropism inducing a lifelong latent infection in humans (Ablashi et al., 2014; Eliassen et al., 2017). HHV-6A/B replicate preferentially in CD4+ T lymphocytes and utilize distinct cell surface receptors: HHV-6A uses CD46, a regulator of complement activation expressed on all nucleated cells, while HHV-6B uses CD134 (also called OX40), a member of the tumor necrosis factor (TNF) receptor superfamily. HHV-6 infects also CD8+ T lymphocytes, NK cells, astrocytes, microglial cells oligodendrocytes, liver cells, human fibroblasts, epithelial cells, endothelial *in vitro* cells (De Bolle et al., 2005).

Human herpesvirus-7 has a narrow tropism for CD4+ T-cells, where it uses the glycoprotein CD4 for cell entry (Lusso et al., 1994).

Human herpesvirus-6 and HHV-7 are immune-modulating and modify the secretion of chemokines and cytokines, with a significant effect on host immune response (Lusso, 2006; Yoshikawa et al., 2009).

Currently, few studies are available on HHV-6 and HHV-7 infection of Natural killer (NK) cells, probably due to the absence of reliable animal models.

Natural killer cells are able to kill tumor cells and virus-infected cells independently of MHC restriction. Patients lacking NK cells are subject to multiple infections by HHV, evidencing their importance in viral immuno-surveillance *in vivo* (van Erp et al., 2019). Several studies demonstrate NK-cell-dependent protective effects during viral infections (Vidal et al., 2011), with a direct killing of infected target cells and production of cytokines (e.g., interferon (IFN)-γ) (Blanc et al., 2011).

HHV-6A/B can infect NK cells (Rizzo et al., 2017). We have reported that NK cells are permissive to both HHV-6A and HHV-6B viruses establishing a lytic replication. Both viruses affect the expression of miRNAs implicated in NK cell development, maturation and functions (miR-146, miR-155, miR-181, miR-223). Moreover, HHV-6A/6B infections modify the expression of transcription factors, with both species increasing ATF3, JUN, and FOXA2, whereas HHV-6A inducing POU2AF1 decrease, and HHV-6B FOXO1 increase, and ESR1 decrease. HHV-6B evades the elimination of infected cells by suppressing surface expression of ligands for NK cell receptors NKG2D and NKp30 (Schmiedel et al., 2016). Meanwhile, the up-regulation of IL-15 production induced by HHV-6A/B and HHV-7 infection results in NK cell antiviral activity (Atedzoe et al., 1997).

Human herpesvirus-7 U21 protein reduces NK activation and cytotoxicity interacting with the NK cell activating ligand ULBP1 that is rerouted to the lysosomal compartment, and down-regulating the surface expression of the NK activating ligands MICA and MICB (Schneider and Hudson, 2011).

The germline-encoded pattern recognition receptors (PRR) and DNA sensors facilitate the NK cells recognition of pathogens during the initial stages of infection, activating downstream signaling cascades and the secretion of type I IFN and pro-inflammatory cytokines.

Endosomal DNA-sensor Toll-like receptor (TLR)-9 has been shown to recognize microbial DNA and induces the host defense against infections (Kawai and Akira, 2010), such as Human cytomegalovirus (HCMV), Herpes simplex virus (HSV)-1 (Hochrein et al., 2004) and HSV-2 (Lund et al., 2003). The hexamers containing unmethylated CpG (cytosine-phosphate-guanine dideoxynucleotide) motifs are the preferential ligands of TLR9 (Hemmi et al., 2000).

Upon HHV infection, viral DNA or aberrantly localized cellular DNA are recognized by the DNA sensor cyclic GMPAMP (cGAMP) synthase (cGAS) that forms the second messenger 2′3′-cGAMP (Diner et al., 2013). cGAMP interacts with the endoplasmic reticulum (ER)-resident adaptor protein stimulator of interferon genes (STING) that dimerizes and translocates from the ER to the Golgi apparatus (Dobbs et al., 2015). Here, Tank-binding kinase 1 (TBK1) is recruited for the interferon regulatory factor 3 (IRF3) phosphorylation. IRF3 dimerizes (Tanaka and Chen, 2012) and translocates into the nucleus, inducing the expression of type I IFN. STING can also recruit Signal transducer and activator of transcription (STAT)6 to the endoplasmic reticulum, where it dimerizes and translocates to the nucleus, inducing target genes involved in immune cell homing, such as chemokines (Chen et al., 2012). Gamma-interferon-inducible protein 16 (IFI16) is a cytosolic DNA sensor (Diner et al., 2013) of the Pyrin and HIN domain (PYHIN) protein family. In the presence of HHV infection, IFI16 translocates to the cytoplasm where it induces STING-mediated signaling (Almine et al., 2017) or synergizes with cGAS as a DNA co-sensor (Almine et al., 2017; Dunphy et al., 2018).

The role of DNA sensors in NK cell anti-HHV-6 and HHV-7 response is unclear and additional studies are needed to understand the biological consequences on pathway signaling. Here, we examine the role of DNA sensors in human NK cells infected by HHV-6 and HHV-7.

## Materials and Methods

### NK Cells

Natural killer 92 (ATCC CRL-2407) cell line was grown in MEM-Alpha medium (Minimal Essential Medium, Gibco BRL, Invitrogen Corporation, Carlsbad, CA, United States) supplemented with 20% of FCS (fetal calf serum, Euroclone, Pero, MI, Italy), 0.1 mM 2-Mercaptoethanol (Gibco BRL, Invitrogen Corporation, Carlsbad, CA, United States), 100 U/mL penicillin, 100 μg/mL streptomycin and 150 U/mL of IL-2. Cell cultures were maintained at 37°C in humidified atmosphere of 5% CO_2_ in air. For stimulation of DNA sensors we used 2′,3′-cGAMP (Sigma-Aldrich, St. Louis, MO, United States) 1 μM for 30 min in digitonin permeabilization buffer (50 mM HEPES, 100 mM KCl, 3 mM MgCl2, 0.1 mM DTT, 85 mM sucrose, 0.2% BSA, 1 mM ATP, 0.1 mM GTP, pH 7.0) (Srikanth et al., 2019).

Human primary NK cells were obtained from the peripheral blood of healthy blood donors. This study was approved by the “Ferrara Ethics Committee” and we collected written informed consent from all subjects. All subjects gave written informed consent in accordance with the Declaration of Helsinki.

Primary NK cells were separated from peripheral blood samples using the negative magnetic cell separation (MACS) system (Miltenyi Biotec, Gladbach, Germany) (Marci et al., 2016). The analysis of purified cell fraction by flow cytometry with CD3-PerCp-Cy5.5, CD56-FITC moAbs (e-Bioscience, Frankfurt, DE), demonstrated that the NK cell content was >90% (data not shown). NK cells were treated with different mRNA sensors or DNA sensors antagonists/inhibitors. We used: ODN 2087 (Miltenyi Biotec), TLR7 and TLR8 antagonist (0.5 μM); TLR3.CI (Calbiochem, Merck, Darmstadt, Germany), TLR3/dsRNA Complex Inhibitor (30 nM); ODN 2088 (Miltenyi Biotec), TLR7, TLR8, TLR9 antagonist (0.5 μM); H-151 (Invivogen; San Diego, CA, United States) STING antagonist (0.5 μM) (Haag et al., 2018); A15117499 (Sigma-Aldrich, Saint Louis, MS, United States) STAT6 inhibitor (100 nM) (Chiba et al., 2009). To confirm the efficacy of mRNA sensors antagonists/inhibitors, we used synthetic agonists. R-848 (Invivogen), a TLR-7/8 agonist (Gorden et al., 2005) (28), was dissolved in dimethyl sulfoxide (Sigma-Aldrich, St. Louis, MO, United States) at a concentration of 10 mM and stored at 4°C. It was used at the concentration of 3 μM (Gorski et al., 2006). Poly I:C (Sigma-Aldrich), an agonist of TLR3, was reconstituted at 2.5 mg/ml at 50°C and re-annealed before storage at −20°C. It was used at the concentration of 25 μg/ml.

### HHV-6A, HHV-6B and HHV-7 Infection

Cell-free virus inocula were obtained as previously described: HHV-6 variant A (strain U1102) was grown in the J-Jhan cell line (Rotola et al., 1998); HHV-6 variant B (strain Z29) and HHV-7 (strain CZ) (Portolani et al., 1995) were grown in the Sup T1 cell line.

RNA cell extraction was performed with the RNeasy kit (Qiagen, Hilden, Germany). The absence of contaminant DNA in the extracted RNA was assured by DNase treatment and control β-actin PCR without retrotranscription reverse transcription (Caselli et al., 2017; Rizzo et al., 2017). The analysis of virus transcripts was preformed by RNA reverse transcription with the RT2 First strand kit (Qiagen, Hilden, Germany) using cDNA aliquots obtained from 200 ng RNA (Menegazzi et al., 1999; Caselli et al., 2012). We used specific primers for HHV-6 or HHV-7 U42 gene, respectively (Caselli et al., 2012). The sequences are reported in Table 1. Each sample was run in duplicate.

**TABLE 1:** U42 primers.

**Gene**	**Primers**
HHV-6 U42 (Mirandola et al., 1998)	Forward 3′ACGATGGACATGGCTTGTTG5′ Reverse 3′ACCTTACAACGGAGACGCC5′
HHV-7 U42 (Menegazzi et al., 1999)	Forward 3′AAGCTGCAAGACGGAGTTGT5′ Reverse 3′AGTATTCCGGTGAAGCACGA5′

We also evaluated the transcription of latent (EBNA1, EBNA-2, LMP1) and lytic (BAL2) genes of Epstein-Barr virus (EBV), that latently infect NK92 cell line. We used specific primers, as previously reported (Isobe et al., 2004). The lymphoblastoid cell line LCL-B95.8 (kind gift of Professor R. Dolcetti) was used as control of EBV gene expression, after viral cycle activation using TPA (12-*O*-tetradecanoylphorbol-13-acetate) (Sigma-Aldrich), used at 20 ng/ml.

### Immunofluorescence Assay

Human herpesvirus-6 gp116 and HHV-7 KR4 late antigens’ expression was analyzed by immunofluorescence with anti-gp116/64/54 FITC antibody (Ab) (Clone 6A5) (Santa Cruz, United States) or KR4 (a kind gift of HHV-6 foundation), as previously described (Caselli et al., 2012). STING/STAT expression was evaluated with anti-STING PE Ab (Clone T3-680) (BD Biosciences, Italy) and anti-STAT6 FITC (Clone D-1) (Santa Cruz, United States).

### RNA and DNA Sensor mRNA Analysis

Toll-like receptor 3, TLR7, and TLR8 mRNA were analyzed using the set of primers: TLR3 (F:5′-GAGGCGGGTGTTT TTGAACTAGAA-3′, R:5′-AAGTCAATTGTCAAAAATAGG CCT-3′) (Menager et al., 2009); *TLR7* (F:5′-AGTGTCTAAA GAACCTGG-3′, R:5′-CTTGGCCTTACAGAAATG-3′); and *TLR8* (F:5′-CAGAATAGCAGGCGTAACACATCA-3′,R:5′ATG TCACAGGTG CATTCAAAGG -3′) (Hart et al., 2005).

Toll-like receptor 9 and STING mRNA were analyzed using the set of primers: TLR9 5′-CCGTGACAATTACCTGGC CTTC-3′ (forward) and 5′-CAGGGCCTTCAGCTGGTTTC-3′ (reverse) (Bao et al., 2016); STING: Fw: 5′-GCTGCTG TCCATCTATTTCTACT-3′ (forward) and 5′-GCCGCAGATAT CCGATGTAATA-3′ (reverse) (Gram et al., 2017). Actin was used as house-keeping gene and was analyzed with the set of primers: 5′-GATGGAGTTGAAGGTAGTTT-3′ (forward) and 5′- TGC-TATCCAGGCTGTGCTAT-3′ (reverse) (Rizzo et al., 2017).

### Cytokine mRNA Analysis

Uninfected or HHV infected NK92 cells were stimulated with CpG 25 μg/ml CpG (ODN 2006, TIB MOLBIOL) ml 3d.p.i., and the cells were collected 24 h after stimulation. Cytokine mRNAs were analyzed with Real-Time PCR assays for human genes: IFN-alpha: Hs03044218-g1; IL-6: Hs00174131; IL-8: Bt03211906; IL-22: Hs01574154; TNF-alpha: Hs02621508 (Applied Byosystems; ThermoFisher Scientific; United States).

### Cytokine/Chemokine Enzyme Immunosorbent Assay

Levels of CCL3, CCL4, CCL5, IL-4, IL-8, IL-10, IL-13, IFN-alpha, TNF-alpha, IL-8, IFN-gamma were assessed in duplicates in cell culture supernatants using commercial human specific enzyme-linked immunosorbent assays (ELISAs) (myBiosource, United States) following manufacturer’s protocols.

### Western Blot Analysis

Whole cell lysates were prepared by using the RIPA buffer containing proteinase inhibitor cocktail (Sigma-Aldrich, St. Louis, MO, United States). Proteins were quantified by means of the Bradford assay (Bio-Rad; Segrate, MI, Italy) using bovine albumin (Sigma-Aldrich) as standard. Twenty μg of total proteins were loaded in each well and evaluated in denaturating conditions in 10% TGX-Pre-cast gel (Bio-Rad), with subsequent electroblotting transfer onto a PVDF membrane (Millipore, MA, United States). The membrane was incubated with a specific antibody for the protein to be analyzed, then with a horseradish peroxidase (HRP)-conjugated anti-mouse antibody (1:5000; Amersham Biosciences, NJ, United States) and developed with the ECL kit (Amersham Biosciences, NJ, United States). The images were acquired by Geliance 600 (Perkin Elmer, MA, United States). The specific antibodies used were: anti-Myd88 (Clone 4D6), anti-STING (Clone TMEM173), anti-cGAS (CL3605), anti-IFI16 (Clone 2E3), anti-TBK1 (Clone 108A429), anti-IRF3 (Clone SD2062), anti-STAT6 (Clone 177C322.1) (Novus Biologics; Italy), anti-IRF3 Ser396 (Clone 4D4G), and anti STAT6 Tyr641 (Clone D859Y) (Cell signaling Technology; United States). The complete Western Blots are reported in Supplementary Figures S2, S3.

### Intracellular TLR9 Expression by Flow Cytometry

Intracellular expression of TLR9 was quantified fixing NK92 cells with 2% formaldehyde and permeabilizing them for intracellular staining with anti-TLR9-PE (Clone anti-GJ15A7) (BD Biosciences). Cells were analyzed by flow cytometry (FACS Canto II, BD) and FlowJo software (Tree Star Inc). Viable cells were assessed by propidium iodide. Approximately 10^5^ cells were collected for each individual sample.

### Statistical Analysis

Since the biological variables presented a normal distribution (Kruskal–Wallis test, *p* > 0.05), they were evaluated by Student *t* test by Graph pad software. A *p*-value < 0.05 was defined statistically significant.

## Results

### HHV-6A, HHV-6B and HHV-7 Infect NK Cells

**FIGURE 1:**
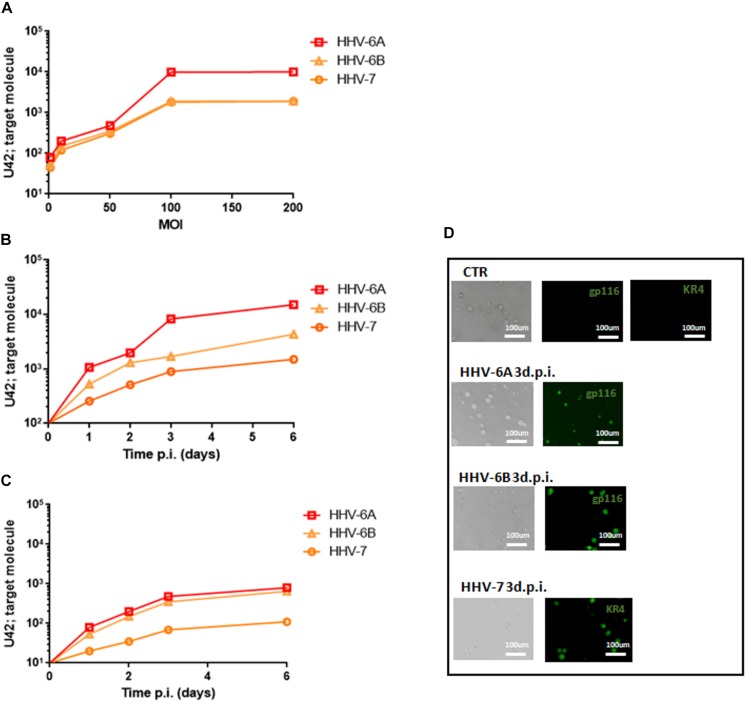
NK92 infection by HHV-6A, HHV-6B and HHV-7. **(A)** NK92 cells were infected with HHV-6A, HHV-6B or HHV-7 at different multiplicity of infection (1.0, 10.0, 50.0, 100.0, 200.0 m.o.i.). The virus presence (U42 DNA) was evaluated 3 days post infection (d.p.i.). Virus **(B)** presence (DNA) and **(C)** transcription (RNA) were evaluated, respectively, by qPCR and RT-qPCR performed on U42 virus gene, at 1, 2, 3, and 6 d.p.i., as already detailed. The infection was performed with 100 m.o.i. **(D)** HHV-6A and HHV-6B infected NK92 cells were characterized by immunofluorescence for gp116 (late viral protein) expression 3d.p.i. HHV-7 infected NK92 cells were characterized by immunofluorescence for KR4 (late viral protein) expression 3d.p.i. Uninfected NK92 cells (CTR) were used as control. Images were taken in bright field (*left panels*) or fluorescence (*right panels*) (Nikon Eclipse TE2000S) equipped with a digital camera. Original magnification 20×.

As previously reported, HHV-6A and HHV-6B viruses can infect NK cells (Rizzo et al., 2019). Here we showed that NK92 cells are permissive to HHV-6A, HHV-6B, and HHV-7, with a high viral amount 3 days post infection (d.p.i.) using 100 multiplicity of infection (m.o.i.) (Figure 1A). In Figures 1B,C, the expression of DNA and mRNA of U42, an immediate early HHV-6 viral gene, increased during the 6 d.p.i. that were evaluated. Similarly, using HHV-7 U42 specific primers, the expression of both DNA and mRNA increased significantly. We selected the time point 3d.p.i. to perform the subsequent experiments, since it coincides with a high viral amount for both HHV-6 and HHV-7. When we looked at viral late antigens at 3 d.p.i., in particular gp116 for HHV-6 and KR4 for HHV-7, we observed their expression (Figure 1D).

Since NK92 cells arbor a latent EBV infection, we wanted to be sure that it did not affect the results observed. The analysis of latent (EBNA1, EBNA-2, LMP1) and lytic (BZLF1) EBV genes showed no mRNA transcription (Supplementary Figure S1), supporting the absence of any confounding effect of EBV latent infection.

### HHV-6A, HHV-6B and HHV-7 Infection Affects TLR9 Expression

To further dissect and understand the modification induced by HHV-6 and HHV-7 infection of NK cells, we performed the analysis on NK92 cell line to have reproducible data not affected by individual differences in NK cell subpopulations. First, we considered the DNA sensor protein TLR9 (Roda et al., 2005). We infected NK cells with HHV-6A, HHV-6B or HHV-7, and evaluated TLR9 mRNA expression at 3 d.p.i. comparing TLR9 mRNA levels in HHV infected cells with those in uninfected NK92 cells.

**FIGURE 2:**
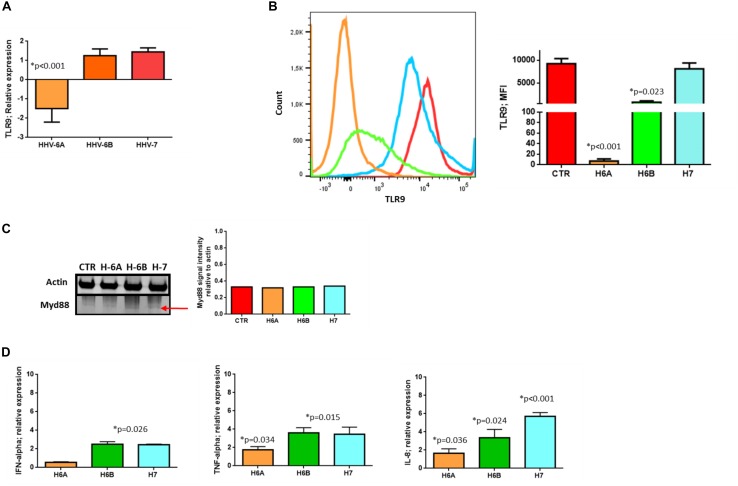
TLR9 analysis. TLR9 **(A)** mRNA relative expression and **(B)** endosomal protein expression in uninfected, HHV-6A, HHV-6B or HHV-7 infected NK92 cells 3 d.p.i. NK92 cells were intracytoplasmic stained for anti-TLR9-PE (Clone anti-GJ15A7). Representative histograms are reported. The histograms showed the MFI (mean fluorescence intensity) values of three independent experiments. ^∗^*p* values Student *t* test. **(C)** Western Blot analysis for house-keeping actin (upper blot) and Myd88 (lower blot, a red arrow indicates the localization of the bands of interest) expression in uninfected (CTR), HHV-6A, HHV-6B or HHV-7 infected NK92 cells 3 d.p.i. The molecular weights were determined by protein ladder (14.4-97.4kDa) (BioRad). Actin was evidenced at 44kDa, Myd88 at 33kDa. The images were acquired by Geliance 600 (Perkin Elmer, MA, United States). The complete Western Blots are reported in Supplementary Figure S2. **(D)** Relative mRNA expression of IFN-alpha, TNF-alpha, IL-8 in HHV-6A, HHV-6B or HHV-7 infected NK92 cells in comparison with uninfected NK92 cells. The cytokines’ mRNA expression was evaluated in NK92 cells 3 d.pi. HHVs infection, 24 h of stimulation with unmethylated CpG DNA motif and with RNA sensor antagonists (ODN 2087 (Miltenyi Biotec), TLR7 and TLR8 antagonist (0.5 μM); TLR3.CI (Calbiochem, Merck, Darmstadt, Germany), TLR3/dsRNA Complex Inhibitor (30 nM).

At mRNA level, we observed that HHV-6A inhibited the expression of TLR9 mRNA and protein (*p* < 0.001; Student *t* test) (Figures 2A,B). HHV-6B and HHV-7 did not affect the TLR9 mRNA expression but we observed a decrease in protein expression after HHV-6B infection (*p* = 0.023; Student *t* test) (Figures 2A,B). When we looked at the TLR9 pathway, we considered the key component Myd88. No modifications were observed in the expression levels of this protein in the NK92 cells infected with any of the three viruses (Figure 2C). These data suggest that the decrease in TLR9 protein expression observed in HHV-6A infected NK92 cells is restricted to the TLR9 gene. Since the expression of TLR9 is fundamental for the pathway activation and transcription of IFN-alpha and pro-inflammatory cytokines (e.g., IFN-alpha, TNF-alpha, IL-6, IL-8, and IL-22), we evaluated the cytokines’ mRNA expression by NK92 cells 3 d.pi. HHVs infection and 24 h of stimulation with unmethylated CpG DNA motif, the ligand for TLR9 activation. We observed that NK92 cells express TLR3, TLR7, and TLR8 mRNA sensing molecules (Supplementary Figure S2B). To be sure that their activation does not affect the results obtained, we inhibited them with specific antagonists (ODN2087: TLR7, TLR8 antagonist; TLR3.CI: TLR3 antagonist). The efficacy of RNA sensors’ antagonists was demonstrated by activating the cells with the corresponding agonists (Supplementary Figure S2C). We used synthetic agonists, R-848 (Invivogen), a TLR-7/8 agonist and Poly I:C (Sigma-Aldrich), an agonist of TLR3. We tested the relative mRNA expression of type I interferon (IFN-alpha) (Tabeta et al., 2004; Uematsu and Akira, 2007), as marker of TLR3, TLR7, TLR-8 activation in NK92 cells treated with TLR7/8, TLR3 agonists with or without antagonists treatment. We observed an increase in IFN-alpha levels in TLR7/8, TLR3 agonists treated cells, while the antagonist treatment inhibits the induced expression of IFN-alpha (Supplementary Figure S2C). We showed an increase in IFNA-alpha, TNF-alpha, and IL-8 expression in HHV-6B and HHV-7 infected NK92 cells (Figure 2D), with the highest levels reached by IL-8 with HHV-7 infection (*p* < 0.001; Student *t* test). On the contrary, TNF-alpha and IL-8 expression was slightly modified in HHV-6A infected NK92 cells (Figure 2D). IL-6 and IL-22 were not induced by the viruses (data not shown).

### HHV-6A, HHV-6B and HHV-7 Infection Affects STING Expression and Activation Pathway

**FIGURE 3:**
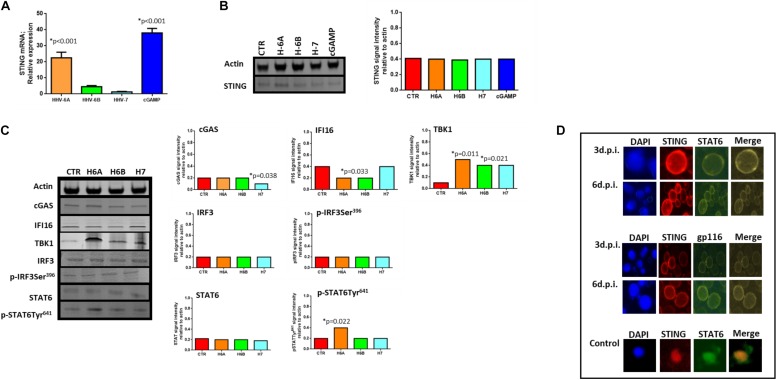
STING pathway analysis. **(A)** STING mRNA relative expression in uninfected (CTR), HHV-6A, HHV-6B or HHV-7 infected NK92 cells 3d.p.i. ^∗^*p* values Student *t* test; **(B)** Western Blot analysis for house-keeping actin (upper blot) and STING (lower blot) expression in uninfected (CTR), HHV-6A, HHV-6B or HHV-7 infected NK92 cells 3d.p.i. For stimulation of the cytoplasmic DNA sensing pathways, we used 2′,3′-cGAMP. The molecular weights were determined by protein ladder (14.4-97.4kDa) (BioRad). Actin was evidenced at 44kDa, STING 35kDa. The images were acquired by Geliance 600 (Perkin Elmer, MA, United States). The complete Western Blots are reported in Supplementary Figure S3. ^∗^*p* value Student *t* test. The histogram represents the STING band intensity in relation with the corresponding actin band. **(C)** Western Blot analysis for house-keeping actin (upper blot) and cGas, IFI16, TBK1, IRF3, pIRF3Ser396, STAT6, pSTAT6Tyr641 (lower blots) expression in uninfected (CTR), HHV-6A, HHV-6B or HHV-7 infected NK92 cells 3 d.p.i. The molecular weights were determined by protein ladder (25-250kDa; 14.4-97.4 kDa). Actin was evidenced at 44kDa, cGas at 68kDa, IFI16 at 100kDa, TBK1 at 80kDa, IRF3 at 55kDa, STAT6 at 120kDa. The histogram represents the STING band intensity in relation with the corresponding actin band. ^∗^*p* value Student *t* test. The images were acquired by Geliance 600 (Perkin Elmer, MA, United States). The complete Western Blots are reported in Supplementary Figure S3. **(D)** HHV-6A infected NK92 cells were characterized by immunofluorescence for STING [anti-STING PE Ab (Clone T3-680)], STAT6 [anti-STAT6 FITC (Clone D-1)] and gp116 (Clone 6A5) expression. (Nikon Eclipse TE2000S) equipped with a digital camera. Original magnification 100×. Uninfected NK92 cells were used as control.

We then considered the DNA sensor protein STING. The stimulation of the cytoplasmic DNA sensing pathways was performed with 2′,3′-cGAMP. At mRNA level, we observed increased levels of STING expression during HHV-6A infection and cGAMP treatment (*p* < 0.001; Student *t* test), while HHV-6B and HHV-7 did not affect STING mRNA expression (Figure 3A). When we considered protein expression, we did not observe any induction of STING expression in all the four conditions (Figure 3B).

When we looked at STING pathway, we considered the key components cGAS and IFI16 as up-stream regulators of STING activation, TBK1 and IRF3 for the NFkB activation pathway and STAT6 for the STAT6-dependent pathway.

cGAs protein expression was not modified by HHV-6A and HHV-6B and only slightly decreased by HHV-7 (*p* = 0.038; Student *t* test). IFI16 protein expression was not modified by HHV-7, while it was down-modulated by HHV-6A and HHV-6B infection (*p* = 0.033; Student *t* test) (Figure 3C). NK92 cell infection induced TBK1 expression (Figure 3C), mainly after HHV-6A infection (*p* = 0.011; Student *t* test). Notably, the Western Blot of TBK1 presented the control lane with a lighter background in comparison with the HHV lanes. We hypothesize that TBK1 moAb might cross-reacts with some HHV proteins and creates a darker background. IRF3 protein expression and Ser396 phosphorylation were not up-regulated by the three viruses. On the contrary, STAT6 Tyr641 phosphorylation was induced by HHV-6A (Figure 3C) (*p* = 0.022; Student *t* test). HHV-6B and HHV-7 did not induce STAT6 expression (Figure 3C). The peculiar activation of STING/STAT6 pathway in HHV-6A infected NK92 cells seems to be confirmed by the different cellular localization of STING and STAT6 (Figure 3D). We observed that already after 3d.p.i. STING and STAT6 co-localized in the peri-nuclear/cytoplasmic region of the HHV-6A infected NK92 cells, that express the late gp116 viral antigen (Figure 3D). On the contrary, the localization of STING and STAT6 in control NK92 cells was prevalently cytosolic (Figure 3D, lower panel).

### HHV-6A, HHV-6B and HHV-7 Infection Affects NK Cell Cytokines/Chemokines Secretion

The activation of DNA sensor proteins is responsible for the expression of cytokines and chemokines (Diner and Cristea, 2015). We evaluated the effect of the activation of the different pathways (TLR9, STING, STAT6) due to viral infections on the cytokine and chemokine expression by primary NK cells. We purified NK cells from peripheral blood samples of five control subjects and infected them with 100 m.o.i. for 3 days (Supplementary Figure S4), in the presence of mRNA sensor molecules antagonists/inhibitors (ODN2087: TLR7, TLR8 antagonist; TLR3.CI: TLR3 inhibitor) differently combined with DNA sensor molecules antagonists/inhibitors (ODN2088: TLR9 antagonist; H151: STING antagonist; AS1517499: STAT6 inhibitor).

**FIGURE 4:**
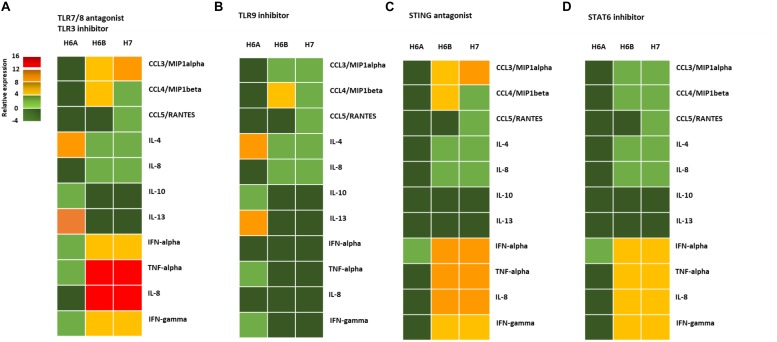
NK cell cytokine/chemokine protein amount. The concentrations of cytokines in the culture supernatants of HHV-6A, HHV-6B or HHV-7 infected primary NK cells 3d.p.i. were quantified by immunoassays (CCL3, CCL4, CCL5, IL-4, IL-8, IL-10, IL-13, IFN-alpha, TNF-alpha, IL-8, IFN-gamma) (myBiosource, United States. The data are presented as relative to the expression of uninfected primary NK cells. Five donors have been analyzed and the data are the mean of the results obtained. The cell were treated with RNA/DNA sensor antagonists/inhibitors: **(A)** ODN 2087 TLR7 and TLR8 antagonist (0.5 μM) + TLR3.CI TLR3/dsRNA Complex Inhibitor (30 nM); **(B)** TLR3.CI TLR3/dsRNA Complex Inhibitor (30 nM) + ODN 2088 (Miltenyi Biotec), TLR7, TLR8, TLR9 antagonist (0.5 μM); **(C)** ODN 2087 TLR7 and TLR8 antagonist (0.5 μM) + TLR3.CI TLR3/dsRNA Complex Inhibitor (30 nM) + H151 STING antagonist (0.5 μM); **(D)** ODN 2087 TLR7 and TLR8 antagonist (0.5 μM) + TLR3.CI TLR3/dsRNA Complex Inhibitor (30 nM) + AS1517499 STAT6 inhibitor (100 nM). The green squares are representative of slight differences that do not reach the a significant statistical value.

We observed a different cytokine/chemokine expression during viral infections in the presence of different inhibitors. In the presence of mRNA sensor molecules antagonists/inhibitors, HHV-6A infected NK cells up-regulated IL-4 and IL-13 and slightly induced IL-10, TNF-alpha, IFN-alpha, and IFN-gamma (Figure 4A). NK cells infected with HHV-6B and HHV-7 up-regulated CCL3, IFN-alpha, TNF-alpha, IL-8, and IFN-gamma and slightly induced IL-4 and CCL4.

When we added the TL9 antagonist ODN2088, we observed no more induction in TNF-alpha, IFN-alpha, IL-8, and IFN-gamma in HHV-6A infected NK cells (Figure 4B). The TLR9 antagonists drastically reduced the secretion of CCL3, IFN-alpha, TNF-alpha, IL-8, and IFN-gamma in HHV-6B and HHV-7 infected NK cells (Figure 4B). The addition of STING antagonist H151 reduced cytokine/chemokine secretion by HHV-6A infected NK cells, maintaining only IFN-alpha levels unaltered (Figure 4C). IN HHV-6B and HHV-7 infected NK cells there was only a slight reduction in IFN-alpha, TNF-alpha, and IL-8 secretion (Figure 4C). The addition of STAT6 inhibitor AS1517499 in HHV-6A infected NK cell cultures resulted in a similar reduction of cytokine/chemokine secretion observed with the STING antagonist H151 (Figure 4D). AS1517499 slightly reduced the secretion of CCL3, CCL4, TNF-alpha, and IL-8 in HHV-6B and HHV-7 infected NK cells (Figure 4D).

## Discussion

Here, we evaluated the effect of HHV-6A, HHV-6B, and HHV-7 NK cell infection on DNA sensor molecules. We used, for the first set of experiments, NK92 cell line to avoid individual differences in NK cell subpopulations. The confirmatory experiments were performed on primary NK cells obtained from control subjects. We found that the three viruses affect the DNA sensors in NK cells differently.

TLR 9 mRNA and protein levels were inhibited by HHV-6A infection but no effect was observed in the protein expression of Myd88, a downstream mediator of the TLR9 pathway. These data suggest a particular impairment in the TLR9 pathway during HHV-6A infection, that is slightly evident only during HHV-6B but not HHV-7 infection. As a proof of concept, the analysis of the TLR9 down-stream genes’ activation (Huang and Yang, 2010), in the presence of mRNA sensor molecules (TLR3, TLR7, TLR8) antagonists/inhibitors, showed that HHV-6B and HHV-7 infections induced an increase in TNF-alpha and IL-8 expression, with the highest levels reached by IL-8 during HHV-7 infection. On the contrary, HHV-6A infection slightly modified TNF-alpha and IL-8 expression. TLR9 signaling is essential for the early cytotoxicity of NK cells during infections (Liese et al., 2007). The reduction of TLR9 expression in HHV-6A infected NK cells leads to an impaired cytokine expression that might prevent NK cells activation toward target cells and slow down the inflammatory response needed to fight the infection. On the contrary HHV-6B and HHV-7 induced TNF-alpha and IL-8 secretion by NK cells, as previously reported for astrocyte cultures (Chi et al., 2012), where HHV-6 infection promoted transforming growth factor β (TGF-β), IL-6, IL-8, and TNF-alpha production. During HHV-6B and HHV-7 infection, we observed a high expression of IL-8, as previously reported in HHV-6 infected Hep G2 liver cells (Inagi et al., 1996), where a significant induction of IL-8 gene expression was observed. These data suggest that HHV-6B and HHV-7 may induce a cytokine-mediated inflammatory response infecting NK cells. On the contrary, HHV-6A reduced pro-inflammatory cytokine release, which could result in NK cell dysfunction *in vivo*.

STING mRNA expression was increased during HHV-6A infection, but not during HHV-6B and HHV-7 infection. Strangely, STING protein levels were not modified by all the three viruses, suggesting that the over-production of STING mRNA during HHV-6A infection is degraded, probably to convey the transduction machinery to the viral mRNAs. Similarly, 2′,3′-cGAMP affected only mRNA but not protein expression. Since STING is already efficiently expressed, we can hypothesize that 2′,3′-cGAMP affects the transcription of mRNA that is then degraded.

When we looked at STING pathway, we observed that TBK1 protein expression is induced by all three viruses, but without an enhanced IRF3 Ser^396^ phosphorylation. On the contrary, STAT6 Tyr^641^ phosphorylation was induced only by HHV-6A with a perinuclear co-localization of STING and STAT6 at 3 d.p.i. in HHV-6A infected cells, as suggested by the co-localization with the gp116 late antigen. It has been already shown that viruses or cytoplasmic nucleic acids trigger STING to recruit STAT6 to the ER, where it is phosphorylated on Ser^407^ by TBK1 and on Tyr^641^ by IL-4/IL-13 pathway (Chen et al., 2003). Dimerized STAT6 then translocates to the nucleus where it induces target genes responsible for immune cell homing. We have evaluated only STAT6 Tyr^641^ phosphorylation since there are no commercial antibodies available toward Ser^407^. However, since TBK1 is induced in HHV-6A infected NK92 cells, we can hypothesize that both Ser^407^ and Tyr^641^ might be phosphorylated in HHV-6A infected NK92 cells and lead to down-stream genes’ transcription. As a proof of concept, Atf3 transcription factor, that we have previously found up-modulated by HHV-6A infection of NK cells, is induced by IL-4 through STAT6 (Chen et al., 2003), supporting the activation of STING/STAT6 pathway.

cGAS protein levels were down-modulated by HHV-7 infection, while HHV-6A and HHV-6B remained at a basal level. IFI16 protein expression was down-modulated by HHV-6A and HHV-6B infection, while it remained at a basal level during HHV-7 infection. These results might confirm previous papers on HSV-1 that induced the degradation of IFI16 by a proteasome and apparently ICP0-dependent mechanism (Orzalli et al., 2012). Similarly, HHV-6A and HHV-6B seem to maintain the cGAS up-stream activation of STING pathway. On the contrary, HHV-7 data support the role of IFI16 as the primary HHV DNA sensor and restriction factor (Gariano et al., 2012), where cGAS has an indirect role in the presence of nuclear HHV DNA by interacting and stabilizing IFI16 (Orzalli et al., 2015).

The analysis of cytokines/chemokines secretion in HHV infected primary NK cells, showed a different behavior in the presence of the different viruses. The NK cell response seem to be similar in the presence of both HHV-6B and HHV-7 viral infection. NK cells express mainly CCL3, IFN-alpha, TNF-alpha, IL-8, and IFN-gamma. The use of DNA sensors antagonists assigns to TLR9 the main effect on cytokines/chemokines expression. On the contrary, HHV-6A infection of NK cells induced IL-4, IL-10, and IL-13. The addition of antagonists/inhibitors against STING and STAT6 reduced drastically the secretion of these cytokines by NK cells, supporting the activation of STING/STAT6 pathway as predominantly implicated in the response of NK cells to HHV-6A infection. These results suggest a chemoattractant role for cytokine/chemokine secreted by HHV-6B and HHV-7 infected NK cells, while HHV-6A infected NK cells showed a viral-driven Th2 response (Kaiko et al., 2010).

Collectively, these results show an implication of TLR9 DNA sensor in the cytokine/chemokine expression by NK cells infected with HHV-6B and HHV-7. These might lead to the active control of *in vivo* viral spreading. HHV-6A infected NK cells conversely induced STING/STAT6 pathway, as a mechanism of anti-viral activation, but were characterized by a Th2 type response, providing a potential new mechanism used by HHV-6A to induce immunosuppression and immune evasion (Horan et al., 2013; Christensen and Paludan, 2017). To confirm these results, further studies are needed, to dissect the viral mechanism that leads to differential response of NK cells in the presence of different HHV infections.

In conclusion, we have shown that HHV-6A, HHV-6B, and HHV-7 infection of NK cells interact differently with cellular DNA sensors. Strikingly, HHV-6B behaves similarly to HHV-7 compared to HHV-6A, confirming the difference of HHV-6A and -6B in their molecular, epidemiological and biological properties (Ablashi et al., 2014).

## Data Availability Statement

All datasets generated for this study are included in the article/Supplementary Material.

## Author Contributions

DB and RR analyzed the results and wrote the manuscript. DB performed the DNA sensor and cytokine/chemokine analysis. EC, IS, and MD’A performed viral infection. VG and AR performed viral titration and *in vitro* experiments. IB and RR performed cytofluorimetry. MS performed cell cultures. DD revised the manuscript.

## Conflict of Interest

The authors declare that the research was conducted in the absence of any commercial or financial relationships that could be construed as a potential conflict of interest.

## Supplementary Material

FIGURE S1 EBV mRNA expression of latent (EBNA1, EBNA-2, LMP1) and lytic (BALF2) EBV genes in NK92 cell line. The lymphoblastoid cell line LCL-B95.8 (kind gift of Professor R. Dolcetti) was used as control of EBV gene expression, after viral cycle activation using TPA (12-*O*-tetradecanoylphorbol-13-acetate) (Sigma-Aldrich), used at 20 ng/ml.

FIGURE S2 **(A)** Complete Western Blots for Figure 2C. **(B)** RT-PCR for TLR3, TLR7, and TLR8 expression in NK92 cell line. **(C)** Relative mRNA expression of IFN-alpha in the culture supernatants of NK92 cells untreated or treated with synthetic agonists (R-848, TLR7/8 agonist; Poly I:C, TLR3 agonist) with or without ODN 2087 TLR7 and TLR8 antagonist (0.5 μM) + TLR3.CI TLR3/dsRNA Complex Inhibitor (30 nM).

FIGURE S3 Complete Western Blots for Figures 3B,C.

FIGURE S4 Virus **(A)** presence (DNA) and **(B)** transcription (RNA) were evaluated, respectively, by qPCR and RT-qPCR performed on U42 virus gene, at 1, 2, 3, and 6 d.p.i., as already detailed. The infection was performed with 100 m.o.i. in primary NK cells.
