# Supplementary figures and images for: DNA Sensors’ Signaling in NK Cells During HHV-6A, HHV-6B and HHV-7 Infection
Source: Front Microbiol. 2020 Feb 19;11:226. doi: 10.3389/fmicb.2020.00226 (PMC7042408; doi:10.3389/fmicb.2020.00226)

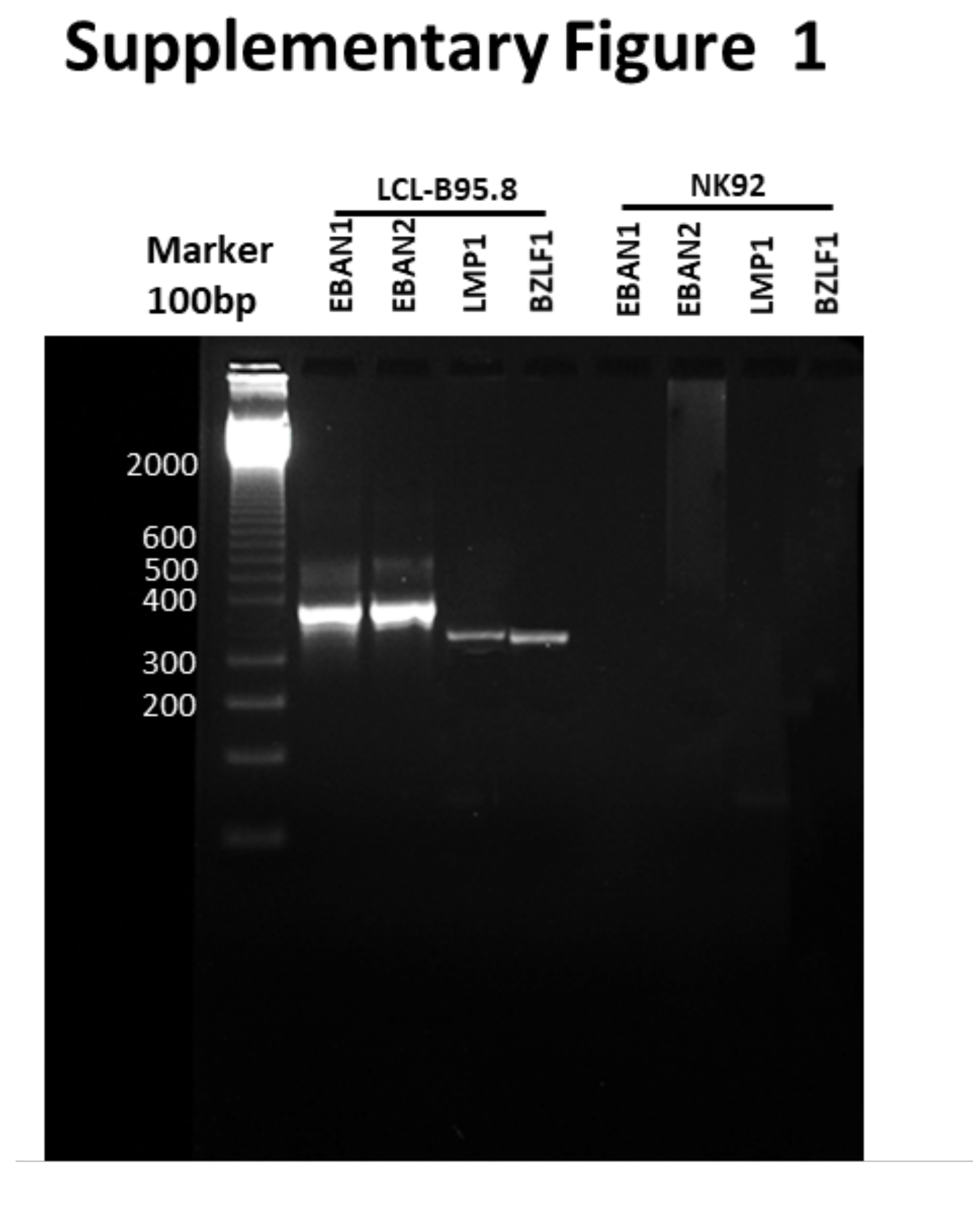

Supplement: FIGURE S1 — EBV mRNA expression of latent (EBNA1, EBNA-2, LMP1) and lytic (BALF2) EBV genes in NK92 cell line. The lymphoblastoid cell line LCL-B95.8 (kind gift of Professor R. Dolcetti) was used as control of EBV gene expression, after viral cycle activation using TPA (12-O-tetradecanoylphorbol-13-acetate) (Sigma-Aldrich), used at 20 ng/ml. [file Image_1.TIFF]

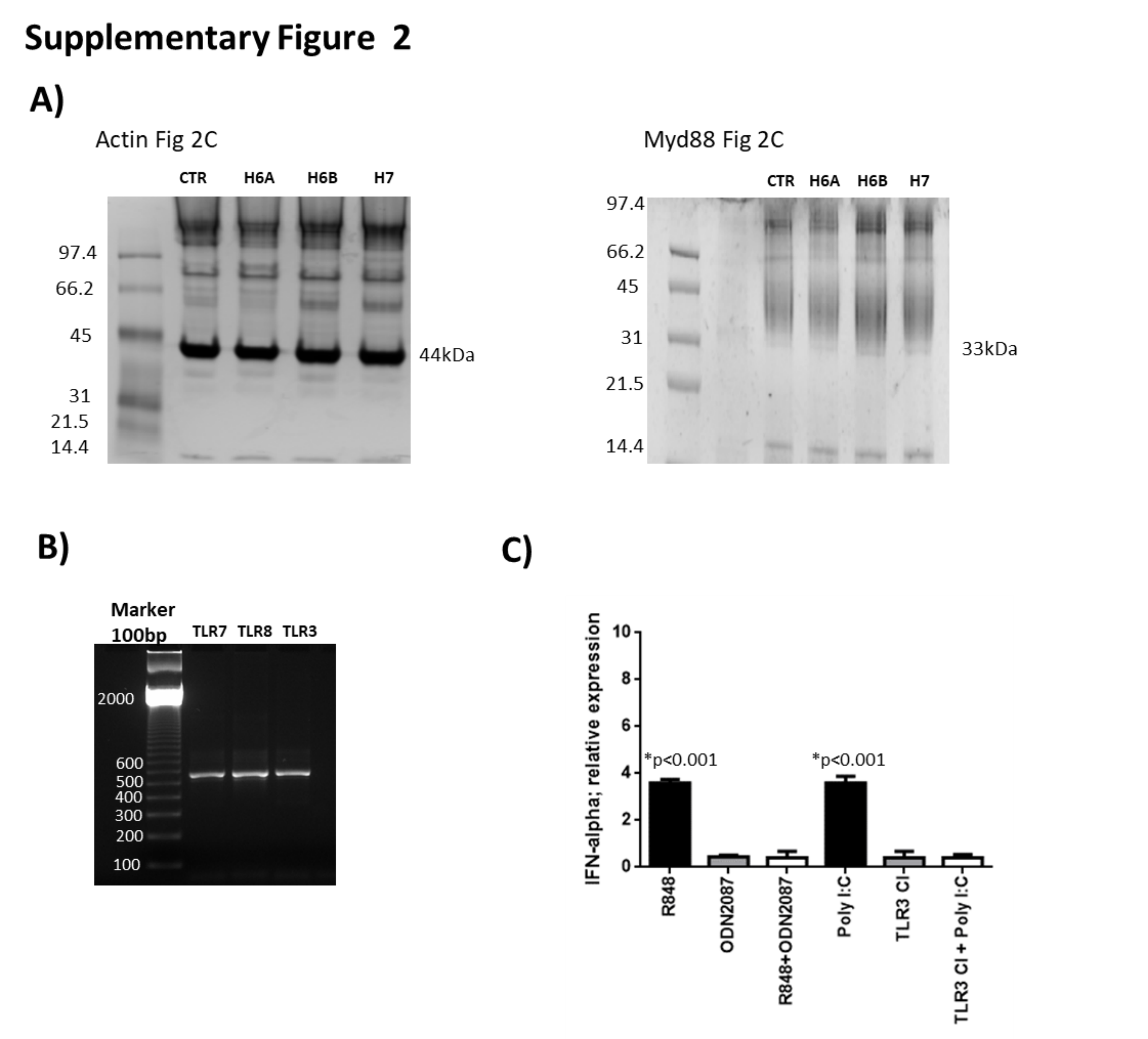

Supplement: FIGURE S2 — (A) Complete Western Blots for Figure 2C. (B) RT-PCR for TLR3, TLR7, and TLR8 expression in NK92 cell line. (C) Relative mRNA expression of IFN-alpha in the culture supernatants of NK92 cells untreated or treated with synthetic agonists (R-848, TLR7/8 agonist; Poly I:C, TLR3 agonist) with or without ODN 2087 TLR7 and TLR8 antagonist (0.5 μM) + TLR3.CI TLR3/dsRNA Complex Inhibitor (30 nM). [file Image_2.TIFF]

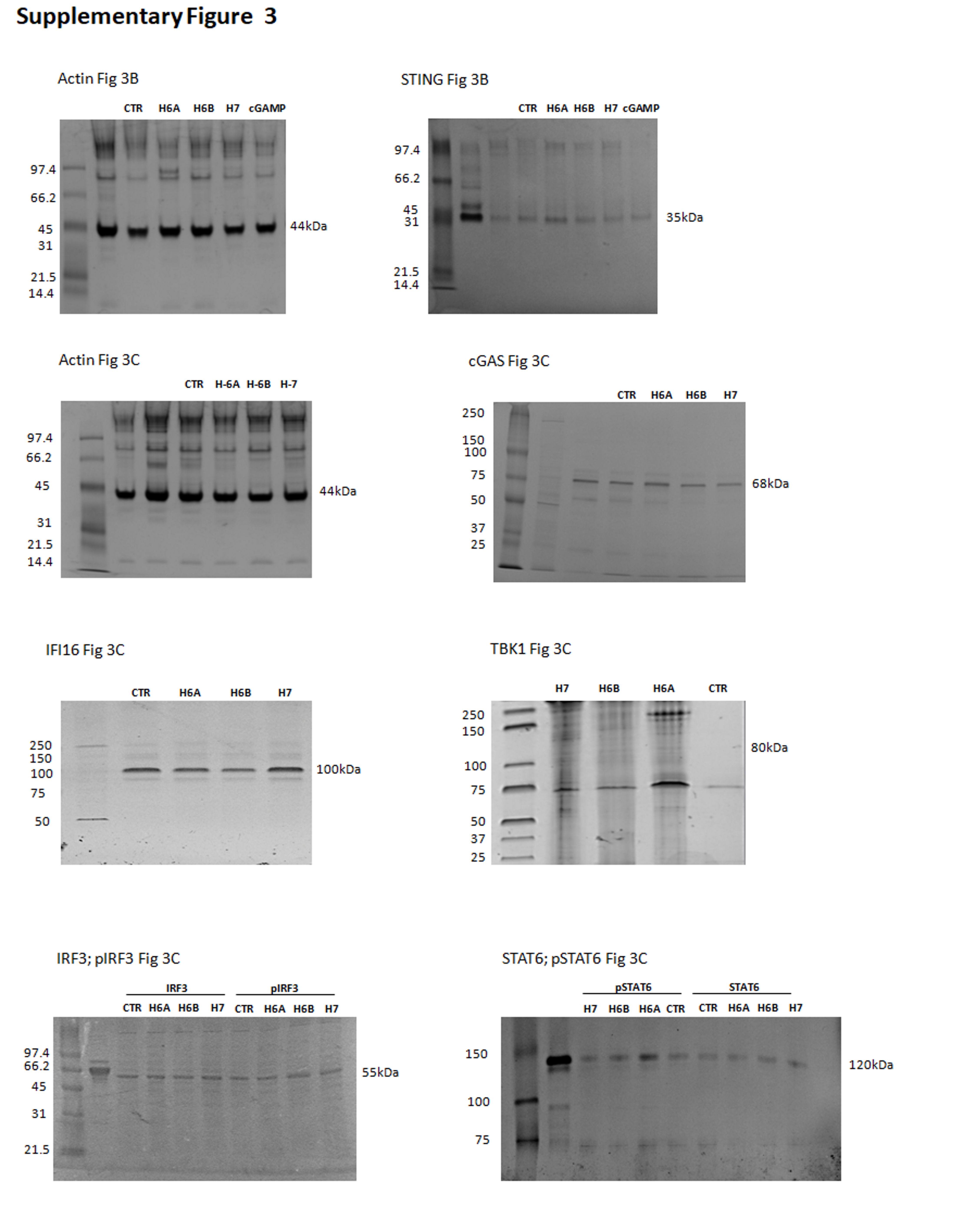

Supplement: FIGURE S3 — Complete Western Blots for Figures 3B,C. [file Image_3.TIF]

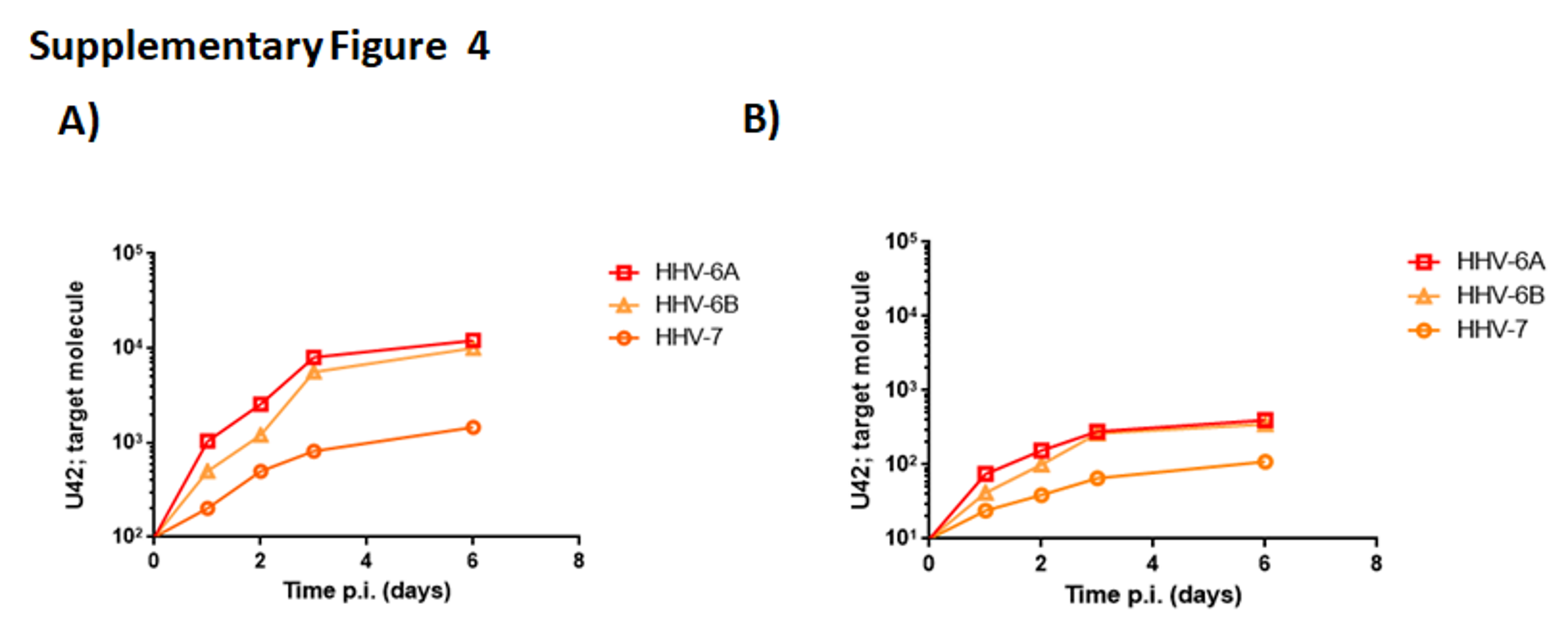

Supplement: FIGURE S4 — Virus (A) presence (DNA) and (B) transcription (RNA) were evaluated, respectively, by qPCR and RT-qPCR performed on U42 virus gene, at 1, 2, 3, and 6 d.p.i., as already detailed. The infection was performed with 100 m.o.i. in primary NK cells. [file Image_4.TIF]
